# Minimum United States Medical Licensing Examination and Comprehensive Osteopathic Medical Licensing Examination Scores Often Do Not Align

**DOI:** 10.7759/cureus.45220

**Published:** 2023-09-14

**Authors:** Dhimitri A Nikolla, Vishnu Mudrakola, Charles J Feronti, Sarah C Bilski, Kaitlin M Bowers

**Affiliations:** 1 Emergency Medicine, Allegheny Health Network, Erie, USA; 2 Emergency Medicine, Summa Health, Akron, USA; 3 Emergency Medicine, Jerry M. Wallace School of Osteopathic Medicine, Campbell University, Lillington, USA

**Keywords:** residency application filters, residency match, nrmp match, comlex-usa, usmle, comprehensive osteopathic medical licensing examination, graduate medical education, united states medical licensing examination

## Abstract

Background

Many residency programs do not accept the Comprehensive Osteopathic Medical Licensing Examination (COMLEX-USA) alone for osteopathic applicants. Furthermore, among those programs that do accept the COMLEX-USA, it is unknown how programs scale their minimum COMLEX-USA scores compared to their minimum United States Medical Licensing Examination (USMLE) scores.

Objective

Our objective was to examine the variation of relative within-program differences between minimum USMLE Step and COMLEX-USA Level scores required for consideration by United States residency programs.

Methods

We performed a cross-sectional analysis of the Fellowship and Residency Electronic Interactive Database Access (FREIDA) database from April 2023, including the 10 specialties with the most training spots in 2022. These specialties were internal medicine, family medicine, pediatrics, emergency medicine, psychiatry, surgery, anesthesiology, obstetrics-gynecology, orthopedic surgery, and neurology. Within-program differences were calculated by subtracting the minimum USMLE Step 1 and 2 scores from the converted minimum USMLE Step 1 and 2 scores calculated from the minimum COMLEX-USA Level 1 and 2 scores using two conversion tools. We present differences as medians with interquartile ranges (IQR). Additionally, we report the proportion of programs with greater than 10-point differences for each step (1 and 2).

Results

Of the 3,364 accredited programs from the examined specialties, we included 1,477 in the Step 1 analysis and 1,227 in the Step 2 analysis with complete data. The median within-program difference between the minimum Step 1 score and the predicted Step 1 score was 12.0 (IQR 2.0, 17.0) using the Barnum and colleagues’ conversion tool and -1.7 (IQR -6.2, 6.3) using the Smith and colleagues’ tool. The median differences for Step 2 were 2.0 (IQR -8.0, 12.0) and -6.5 (IQR -13.9, -1.5) for each tool, respectively. Using the Barnum and Smith conversion tools, 937 (63%) and 435 (29%) programs had a greater than 10-point Step 1 score difference, respectively. Similarly, for Step 2, 564 (46%) and 515 (42%) programs had a greater than 10-point difference with each conversion tool.

Conclusion

There is wide variation in the within-program differences between minimum USMLE and predicted minimum USMLE (from COMLEX-USA) scores. Many programs have greater than 10-point differences, which may be a source of bias in osteopathic applicant selection.

## Introduction

United States (US) postgraduate residency programs often screen applicants using filters in the Electronic Residency Application Service (ERAS) [1,2]. Programs use filters to select competitive applicants and reduce the number of applications for review [3]. Common filters utilized by programs include minimum board examination scores [1,4]. The Comprehensive Osteopathic Medical Licensing Examination (COMLEX-USA) Levels are a series of board examinations required for osteopathic students and graduates to complete training and progress to medical licensure [5]. In addition to the COMLEX-USA, osteopathic applicants are eligible to complete the US Medical Licensing Examination (USMLE) Steps, the series of board examinations required for graduates of US allopathic medical schools [6]. Therefore, for osteopathic applicants, these minimum board examination filters include both the COMLEX-USA Levels and USMLE Steps for those who take it [1,4].

These minimum board score filters create several barriers for osteopathic applicants applying to residency programs. First, although not needed for medical licensure or admittance to residency, many programs expect osteopathic applicants to complete the USMLE to be considered [1]. For example, among 1,095 programs responding to the 2022 National Resident Matching Program (NRMP) Program Director Survey, 56% and 46% required a passing or target score on the USMLE Steps 1 and 2, respectively, for osteopathic applicants [1]. Although this expectation allows programs to compare scores between applicants directly, the prerequisite to complete the USMLE for osteopathic applicants creates a significant financial and opportunity cost for those who choose to complete it [7].

Furthermore, for those osteopathic applicants who choose not to complete the USMLE, the COMLEX-USA scores are scaled differently and are not directly comparable to USMLE scores [8]. Therefore, tools exist for programs to convert COMLEX-USA scores to predicted USMLE scores facilitating a more direct comparison of board examination performance [8-10]. However, these tools produce estimates with wide standard errors [9-11], and they are fundamentally prone to selection bias given that they are derived from osteopathic applicants who took both examinations but are targeted for use on those who do not take the USMLE [8-10].

Nevertheless, COMLEX-USA and USMLE scores correlate well among osteopathic students who take both [8-10,12], and conversion tools are an objective method for programs to compare scores between the two examinations [8-10]. Therefore, COMLEX-USA to USMLE conversion tools are an objective method for programs to select minimum board examination scores that are comparable between the examinations. However, the prevalence of COMLEX-USA to USMLE conversion tool use among postgraduate training programs is unknown. Since the use of these conversion tools would likely result in small within-program differences between the reported minimum USMLE Step scores and predicted minimum Step scores converted from their minimum COMLEX-USA Level scores, our purpose was to examine the variation of these within-program minimum board examination score differences. We hypothesized that within-program minimum board examination score differences would be small given that these conversion tools are freely available and allow programs to compare relative performance between the different board examinations [8-10].

## Materials and methods

Study design and setting

We performed a cross-sectional analysis of the American Medical Association’s Fellowship and Residency Electronic Interactive Database Access (FREIDA) database of residency programs on April 4, 2023. All included programs updated their program information between June 3, 2022 and December 20, 2022, except for 19 of 1,477 (1.3%) programs in the Step 1 analysis and 15 of 1,227 (1.2%) programs in the Step 2 analysis, which had missing last update days.

Participants

We included the 10 medical specialties with the most training spots in 2022, including internal medicine, family medicine, pediatrics, emergency medicine, psychiatry, surgery, anesthesiology, obstetrics-gynecology, orthopedic surgery, and neurology [13]. We only included these larger specialties to limit potential bias from smaller specialties with fewer osteopathic applicants. We excluded programs in the Step 1 analysis that did not report both minimum Step 1 and Level 1 scores and excluded programs in the Step 2 analysis that did not report both minimum Step 2 and Level 2 scores.

Variables

Variables analyzed included minimum board examination scores (Levels 1 and 2, Steps 1 and 2), specialty, program type (university hospital, community hospital, community-based university-affiliated hospital, military-based, other), percent of osteopathic residents (3-year average), and osteopathic recognition. The primary outcome was the within-program differences between the reported minimum USMLE Step scores and predicted minimum Step scores converted from their minimum COMLEX-USA Level scores.

Statistical analysis

Minimum Step scores were compared to minimum predicted Step scores calculated from Level scores using two conversion tools for each Step examination [8-10]. Barnum and colleagues presented a table of Step/Level ranges that align [8]. Therefore, for each minimum Level score, the score was converted to a predicted Step score using a linear scale within each corresponding range. In contrast, Smith and colleagues presented regression formulas that we used to convert each minimum Level score to a predicted Step score [9,10].

We described continuous variables with medians and interquartile ranges (IQR) since all were nonnormal as determined by the Shapiro-Wilk normality test. We described categorical variables with counts and percentages. Confidence intervals (CI) for the median within-program differences between each program’s minimum USMLE and predicted USMLE scores were calculated from the standard deviation (SD) of medians from 1,000 bootstrap resamples. Bootstrap methods have the advantage of not assuming a normal distribution [14]. We examined the variation of within-program differences between minimum Step and Level scores by plotting the within-program differences between minimum Step and minimum predicted Step scores using histograms. Additionally, we reported the proportion of programs with board score differences greater than various thresholds, but in particular, greater than ten points on the USMLE. We felt this threshold was reasonable to identify potential outliers since the NRMP charting outcome reports stratify USMLE scores by ten-point increments, and a difference of ten points would likely impact match success given the known distributions of matched and unmatched senior osteopathic student applicants by the Step score in the included specialties [15]. CIs for the median differences between within-program predicted USMLE scores from the two conversion tools for each Step examination were calculated from the SD of median differences from 1,000 bootstrap resamples.

We did not perform any a priori sample size calculations because our objective was to examine the variation of within-program minimum board score differences and not perform hypothesis testing between reported and predicted USMLE scores. Nevertheless, where reported, CIs were considered significant if they did not include zero. Analyses were performed with R (Version 4.3.0 2023-04-21, R Foundation for Statistical Computing, Vienna, Austria) [16]. Packages and package versions are listed in the appendix. The study protocol was reviewed by Allegheny Health Network Institutional Review Board staff and was not considered human subject research.

## Results

Of the 3,364 programs in the FREIDA database from the included specialties, 1,477 (43.9%) met inclusion criteria in the Step 1 analysis and 1,227 (36.5%) in the Step 2 analysis with complete minimum board score data. The median minimum COMLEX-USA scores were 458 (IQR 400, 500) for Level 1 and 475 (IQR 420, 510) for Level 2. The median minimum USMLE scores were 210 (IQR 200, 220) for Step 1 and 215 (IQR 209, 224) for Step 2. The most common specialty was family medicine and the most common program type was community-based, university-affiliated hospitals (Table 1).

**Table 1:** Program Characteristics IQR, interquartile range; NA, not applicable

	Step 1 Analysis	Step 2 Analysis
n	1,477	1,227
Minimum Board Scores, median (IQR)		
Step 1	210 (200, 220)	NA
Step 2	NA	215 (209, 224)
Level 1	458 (400, 500)	NA
Level 2	NA	475 (420, 510)
Specialty, n (%)		
Anesthesiology	45 (3.0)	35 (2.9)
Emergency medicine	88 (6.0)	66 (5.4)
Family medicine	429 (29.0)	360 (29.3)
Internal medicine	333 (22.5)	304 (24.8)
Neurology	74 (5.0)	61 (5.0)
Obstetrics and gynecology	123 (8.3)	107 (8.7)
Orthopaedic surgery	36 (2.4)	21 (1.7)
Pediatrics	106 (7.2)	71 (5.8)
Psychiatry	144 (9.7)	113 (9.2)
Surgery-general	99 (6.7)	89 (7.3)
Program Type, n (%)		
Community-Based, University-Affiliated Hospital	734 (49.7)	629 (51.3)
Community Hospital	354 (24.0)	316 (25.8)
Military-based	19 (1.3)	17 (1.4)
Other	5 (0.3)	2 (0.2)
University Hospital	365 (24.7)	263 (21.4)
Osteopathic Residents, median (IQR)	25 (9, 47)	26 (10, 49)
Osteopathic Recognition, n (%)		
Yes	116 (7.9)	99 (8.1)
Missing	213 (14.4)	189 (15.4)

**Figure 1:**
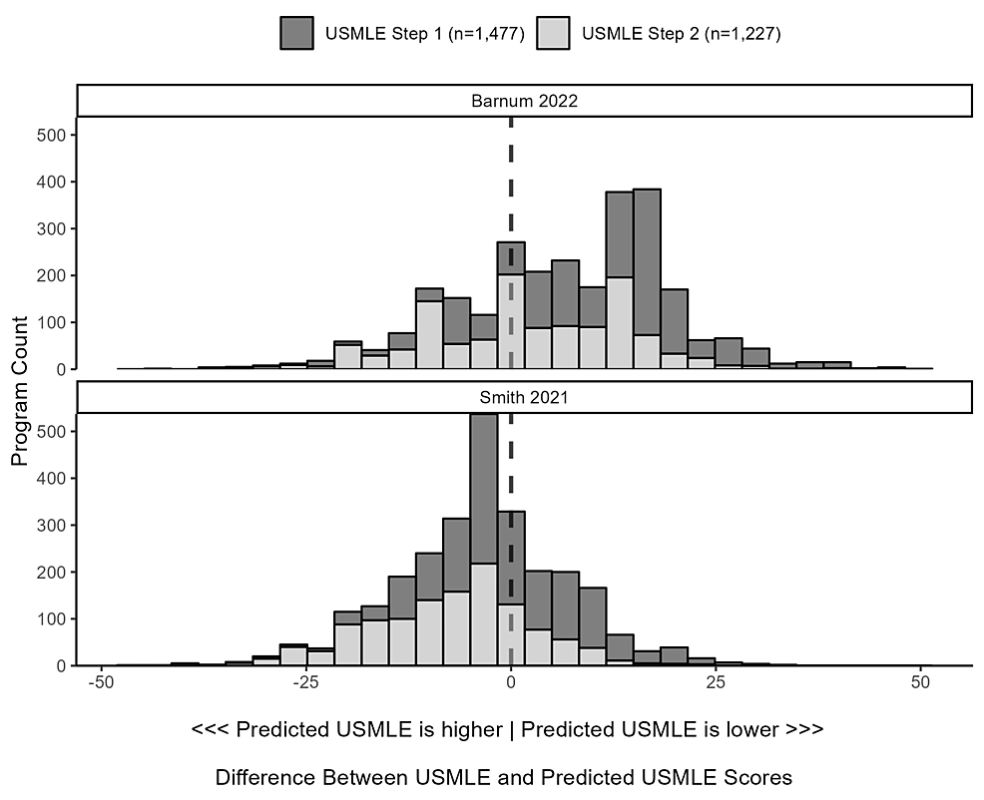
Differences Between the Minimum USMLE Score and Predicted Minimum USMLE Score from the COMLEX-USA Score The histograms display the distribution of within-program differences between minimum USMLE scores and predicted minimum USMLE scores converted from COMLEX-USA scores using two conversion tools. COMLEX-USA, Comprehensive Osteopathic Medical Licensing Examination; USMLE, United States Medical Licensing Examination

**Figure 2:**
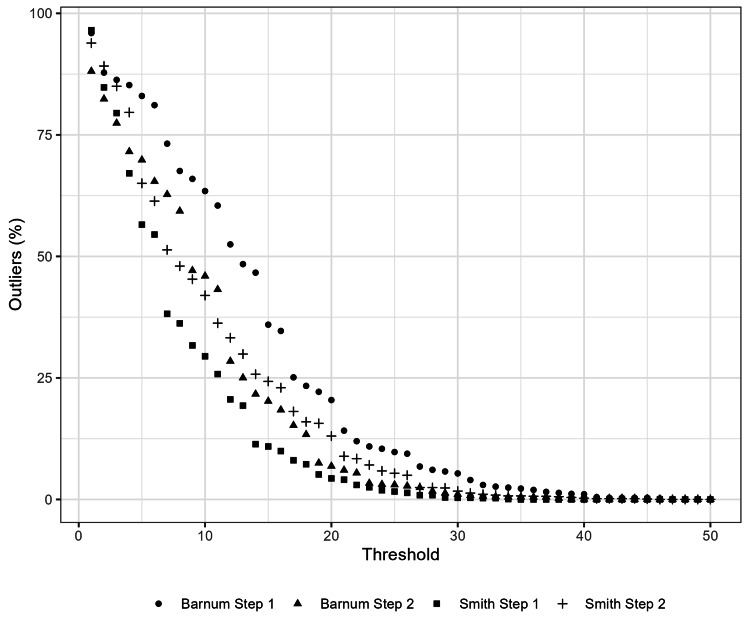
Outliers By Threshold for Within-Program Differences Between Minimum Board Scores The figure displays the proportion of outliers by score threshold for within-program differences between minimum USMLE scores and predicted minimum USMLE scores calculated from the minimum COMLEX-USA scores. USMLE, United States Medical Licensing Examination; COMLEX-USA, Comprehensive Osteopathic Medical Licensing Examination

**Figure 3:**
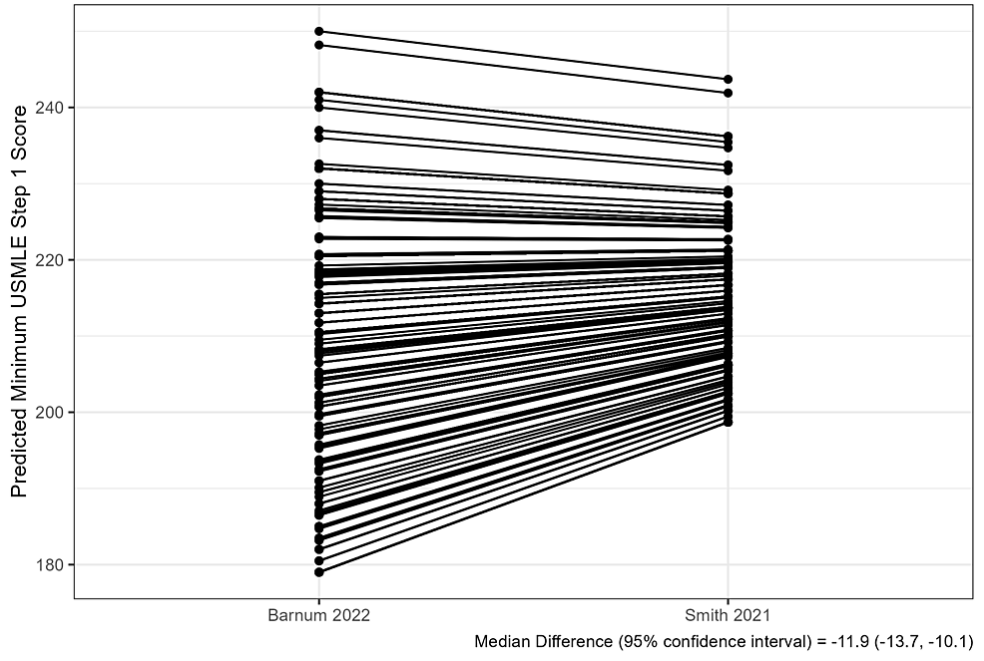
Difference Between COMLEX-USA to USMLE Conversion Tools - Step 1 The figure displays the difference between the predicted minimum USMLE Step 1 scores from the two conversion tools. Lines connect estimates for each program. Bootstrap methods were used to estimate the median difference between the predicted minimum scores for each tool. USMLE, United States Medical Licensing Examination; COMLEX-USA, Comprehensive Osteopathic Medical Licensing Examination

**Figure 4:**
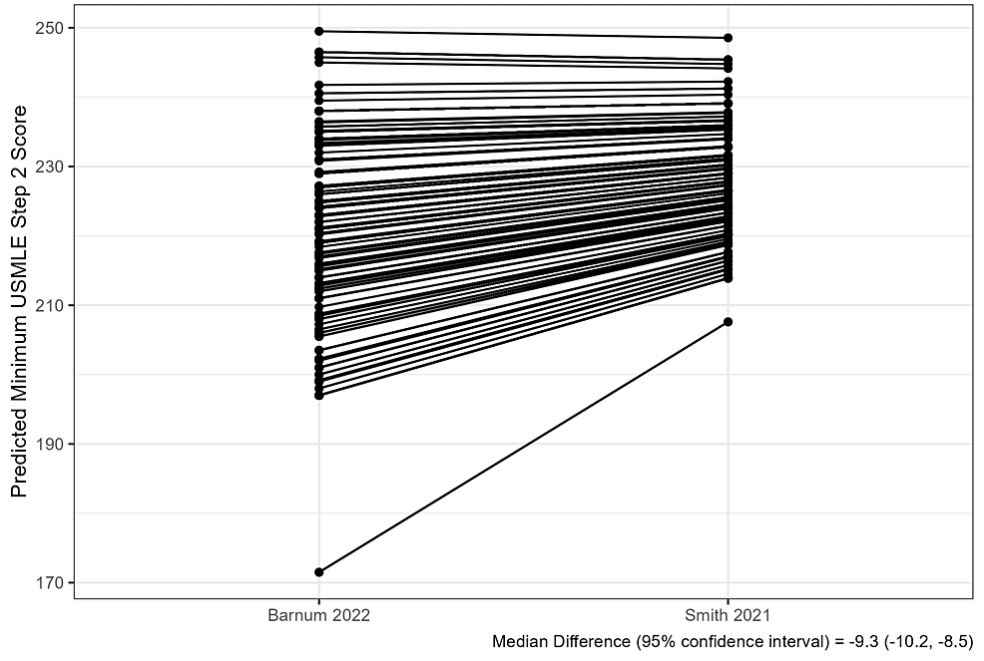
Difference Between COMLEX-USA to USMLE Conversion Tools - Step 2 The figure displays the difference between the predicted minimum USMLE Step 2 scores from the two conversion tools. Lines connect estimates for each program. Bootstrap methods were used to estimate the median difference between the predicted minimum scores for each tool. USMLE, United States Medical Licensing Examination; COMLEX-USA, Comprehensive Osteopathic Medical Licensing Examination

The ranges of within-program differences between minimum USMLE and predicted USMLE scores were wide (Figure 1). The median within-program difference between the minimum Step 1 score and the predicted Step 1 score was 12.0 (IQR 2.0, 17.0) using the Barnum and colleagues’ conversion tool and -1.7 (IQR -6.2, 6.3) using the Smith and colleagues’ tool. The median differences for Step 2 were 2.0 (IQR -8.0, 12.0) and -6.5 (IQR -13.9, -1.5) for each tool, respectively (Table 2). The number of Step 1 outliers (difference >10 points) was 937 (63%) using the Barnum and colleagues’ conversion tool and 435 (29%) using the Smith and colleagues’ tool. The number of Step 2 outliers was 564 (46%) using the Barnum and colleagues’ conversion tool and 515 (42%) using the Smith and colleagues’ tool (Table 2, Figure 2). Predicted minimum USMLE scores differed between the Barnum and Smith conversion tools, with a median difference of -11.9 (95% CI -13.7, -10.1) for Step 1 and -9.3 (-10.2, -8.5) for Step 2 (Figures 3, 4).

**Table 2:** Within-Program Differences Between Minimum USMLE Scores and Predicted USMLE Scores CI, confidence interval; IQR, interquartile Range *The median within-program difference between the minimum USMLE score and the predicted minimum USMLE score calculated from the minimum COMLEX-USA score. **Outliers were programs with score differences greater than ten points.

Conversion Tool	Analysis	Median Within-Program Difference (95% CI)*	IQR of Within-Program Differences (25%, 75%)	Outliers, n / total **	Outliers, % **
Barnum 2022	Step 1	12.0 (11.4, 12.6)	(2.0, 17.0)	937 / 1,477	63
	Step 2	2.0 (0.7, 3.3)	(-8.0, 12.0)	564 / 1,227	46
Smith 2021	Step 1	-1.7 (-2.6, -0.8)	(-6.2, 6.3)	435 / 1,477	29
	Step 2	-6.5 (-6.6, -6.4)	(-13.9, -1.5)	515 / 1,227	42

## Discussion

Within-program differences between the minimum USMLE and predicted minimum USMLE (from COMLEX-USA) scores varied greatly between programs, and the variation differed by conversion tool (Table 2, Figure 1). For example, up to 63% of programs had a minimum board score difference exceeding ten points (Table 2) [15]. However, the median difference between the predicted USMLE scores between the studied conversion tools was about ten for both Steps 1 and 2 (Figures 3, 4); therefore, variable use of different conversion tools between programs may contribute to this variation. Nevertheless, these results suggest that minimum USMLE and COMLEX-USA scores often do not align and have the potential to unfairly influence osteopathic applicant selection in many programs (Figure 1). However, this unfair influence is bidirectional, both favoring and hindering osteopathic applicants without USMLE scores depending on the program (Figure 1).

COMLEX-USA to USMLE conversion tools provide programs with an objective method to establish comparable minimum board scores, facilitating comparisons of academic performance between osteopathic and allopathic applicants [8-10]. However, these conversion tools are less ideal than judging all applicants by their performance on the same examinations [17]. Therefore, many programs require or prefer completion of the USMLE for osteopathic applicants to be considered equally [18,19]. Since these COMLEX-USA to USMLE conversion tools have significant limitations [11] and completion of two similar series of board examinations is needless redundancy [7], more equitable solutions regarding minimum board examination scores are necessary to allow fairness in the match process for osteopathic applicants.

Lastly, these results generate further questions regarding how programs interpret applicant COMLEX-USA scores. The wide variability in differences between minimum USMLE and predicted minimum USMLE scores (converted from COMLEX-USA scores) suggests that there may be wide variability between programs regarding how they interpret the full range of applicant COMLEX-USA scores. Furthermore, this variability in interpreting COMLEX-USA scores may contribute to the lower match success for osteopathic students who do not complete the USMLE [20]. Stakeholders in the match process must work together to find equitable solutions to this variability [17,21].

Limitations

First, many programs do not report both minimum USMLE and COMLEX-USA scores. Therefore, the results of this study cannot be generalized to all residency programs, including programs that do not accept COMLEX-USA alone or do not disclose minimum board score requirements for both examinations. Similarly, programs were more often from family medicine and community-based, university-affiliated hospitals limiting generalizability. Also, this observational data cannot provide causal conclusions about the use of application filters or COMLEX-USA to USMLE conversion tools and match outcomes, nor can it provide definitive evidence for bias in the match process.

We only evaluated the two most recently published COMLEX-USA to USMLE conversion tools; however, older tools exist [22,23]. Therefore, our results may be biased by some programs using older conversion tools. Also, we assessed the conversion tools individually, which may bias the results since not all programs would be expected to use the same conversion tool. Furthermore, we observed significant differences in predicted minimum USMLE scores between the studied conversion tools (Figures 3, 4), suggesting that these tools are not interchangeable. Nevertheless, an ideal COMLEX-USA to USMLE conversion tool does not exist because the tools are derived from candidates who have taken both exams but is intended for use by programs to interpret the COMLEX-USA scores of those osteopathic applicants who have not taken the USMLE [8-10]. Furthermore, osteopathic candidates who complete both examinations perform better on the COMLEX-USA than those who did not take the USMLE, suggesting they are likely more competitive applicants [8]. For example, Barnum and colleagues reported that the mean COMLEX-USA Level 1 score of their study cohort used to derive their tool was 25.4 points greater than the overall Level 1 score [8]. Similarly, their cohort’s mean Level 2 score was 44.0 points greater than the overall mean [8]. However, the impact of these observed within-program minimum board examination score differences may not be practically significant given the fewer applicants that apply to each specialty with scores near common minimum score thresholds [15]. Lastly, Step 1 and Level 1 have transitioned to pass/fail scoring; therefore, it is unknown how this transition may impact the use of minimum board examination scores and scaling of minimum USMLE and COMLEX-USA scores by programs in the future [24].

## Conclusions

There is wide variation in the within-program differences between minimum USMLE and predicted USMLE (converted from COMLEX-USA) scores. These results suggest that within-program differences between minimum USMLE and COMLEX-USA scores may be a source of bias in osteopathic applicant selection.

## Figures and Tables

**Figure 5:**
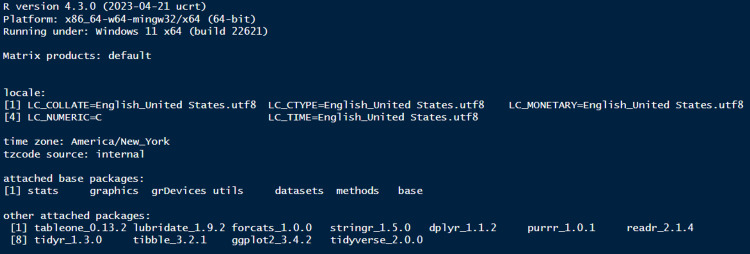
R Version and Packages The figure displays the R version and packages used for the analysis.

## References

[1] (2022). Data Release and Research Committee: Results of the 2022 NRMP Program Director Survey. https://www.nrmp.org/match-data-analytics/residency-data-reports/.

[2] (2023). 2023 ERAS Program Director's Workstation (PDWS) User Guide. https://www.aamc.org/media/55096/download?attachment.

[3] Schrock JB, Kraeutler MJ, Dayton MR, McCarty EC (2017). A cross-sectional analysis of minimum USMLE Step 1 and 2 criteria used by orthopaedic surgery residency programs in screening residency applications. J Am Acad Orthop Surg.

[4] (2020). Data Release and Research Committee: Results of the 2020 NRMP Program Director Survey. https://www.nrmp.org/match-data-analytics/archives/.

[5] Maholtz DE, Erickson MJ, Cymet T (2015). Comprehensive Osteopathic Medical Licensing Examination-USA level 1 and level 2-cognitive evaluation preparation and outcomes. J Am Osteopath Assoc.

[6] Rashid H, Coppola KM, Lebeau R (2020). Three decades later: a scoping review of the literature related to the United States Medical Licensing Examination. Acad Med.

[7] Ahmed H, Carmody JB (2020). Double jeopardy: The USMLE for osteopathic medical students. Acad Med.

[8] Barnum S, Craig B, Wang X (2022). A concordance study of COMLEX-USA and USMLE scores. J Grad Med Educ.

[9] Smith T, Carmody JB, Kauffman M, Gnarra J (2021). Predicting osteopathic medical student performance on the United States Medical Licensing Examination Step 2 clinical knowledge from results of the Comprehensive Osteopathic Medical Licensing Examination Level 2-Cognitive Evaluation. Cureus.

[10] Smith T, Kauffman M, Carmody JB, Gnarra J (2021). Predicting osteopathic medical students' performance on the United States Medical Licensing Examination from results of the Comprehensive Osteopathic Medical Licensing Examination. Cureus.

[11] Lee AS, Chang L, Feng E, Helf S (2014). Reliability and validity of conversion formulas between comprehensive osteopathic medical licensing examination of the United States level 1 and United States medical licensing examination step 1. J Grad Med Educ.

[12] Sandella JM, Gimpel JR, Smith LL, Boulet JR (2016). The use of COMLEX-USA and USMLE for residency applicant selection. J Grad Med Educ.

[13] (2022). Results and Data 2022 Main Residency Match. Data.

[14] Carpenter J, Bithell J (2000). Bootstrap confidence intervals: when, which, what? A practical guide for medical statisticians. Stat Med.

[15] (2022). Charting Outcomes in the Match: Senior Students of U.S. DO Medical Schools. https://www.nrmp.org/match-data-analytics/residency-data-reports/.

[16] (2023). R: A language and environment for statistical computing.. https://www.R-project.org/.

[17] Ahmed H, Carmody JB (2022). COMLEX-USA and USMLE for osteopathic medical students: should we duplicate, divide, or unify?. J Grad Med Educ.

[18] Nikolla DA, Jaqua BM, Tuggle T, Jarou ZJ (2022). Differences between emergency medicine residency programs that accept the Comprehensive Osteopathic Medical Licensing Examination of the United States and those that prefer or only accept the United States Medical Licensing Examination. Cureus.

[19] Heard MA, Buckley SE, Burns B, Conrad-Schnetz K (2022). Identifying attitudes toward and acceptance of osteopathic graduates in surgical residency programs in the era of single accreditation: results of the American College of Osteopathic Surgeons medical student section questionnaire of program directors. Cureus.

[20] Nikolla DA, Stratford CV, Bowers KM (2022). Reported completion of the USMLE Step 1 and match outcomes among senior osteopathic students in 2020. J Osteopath Med.

[21] (2023). FAIR Act Introduced for DO Parity in GME. https://www.nbome.org/news/fair-act/.

[22] Sarko J, Svoren E, Katz E (2010). COMLEX-1 and USMLE-1 are not interchangeable examinations. Acad Emerg Med.

[23] Slocum PC, Louder JS (2006). How to predict USMLE scores from COMLEX-USA scores: a guide for directors of ACGME-accredited residency programs. J Am Osteopath Assoc.

[24] Patel OU, Haynes WK, Burge KG (2022). Results of a National Survey of Program Directors' perspectives on a Pass/Fail US Medical Licensing Examination Step 1. JAMA Netw Open.

